# Relationship between outdoor temperature and cardiovascular disease risk factors in older people

**DOI:** 10.1177/2047487316682119

**Published:** 2016-11-29

**Authors:** Claudio Sartini, Sarah JE Barry, Peter H Whincup, S Goya Wannamethee, Gordon DO Lowe, Barbara J Jefferis, Lucy Lennon, Paul Welsh, Ian Ford, Naveed Sattar, Richard W Morris

**Affiliations:** 1 Department of Primary Care and Population Health, University College London, UK; 2 Institute of Health and Wellbeing, College of Medical, Veterinary and Life Sciences. Robertson Centre for Biostatistics, University of Glasgow, UK; 3 Population Health Research Institute, St George’s University of London, UK; 4 Institute of Cardiovascular and Medical Sciences, University of Glasgow, UK; 5 School of Social and Community Medicine, University of Bristol, UK

**Keywords:** Biomarkers, outdoor temperature, older adults, cardiovascular disease risk factors

## Abstract

**Background:**

Previous studies demonstrated that lower outdoor temperatures increase the levels of established cardiovascular disease risk factors, such as blood pressure and lipids. Whether or not low temperatures increase novel cardiovascular disease risk factors levels is not well studied. The aim was to investigate associations of outdoor temperature with a comprehensive range of established and novel cardiovascular disease risk factors in two large Northern European studies of older adults, in whom cardiovascular disease risk is increased.

**Design and methods:**

Data came from the British Regional Heart Study (4252 men aged 60–79 years) and the Prospective Study of Pravastatin in the Elderly at Risk (5804 men and women aged 70–82 years). Associations between outdoor temperature and cardiovascular disease risk factors were quantified in each study and then pooled using a random effects model.

**Results:**

With a 5℃ lower mean temperature, total cholesterol was 0.04 mmol/l (95% confidence interval (CI) 0.02–0.07) higher, low density lipoprotein cholesterol was 0.02 mmol/l (95% CI 0.01–0.05) higher and SBP was 1.12 mm Hg (95% CI 0.60–1.64) higher. Among novel cardiovascular disease risk factors, C-reactive protein was 3.3% (95% CI 1.0–5.6%) higher, interleukin-6 was 2.7% (95% CI 1.1–4.3%) higher, and vitamin D was 11.2% (95% CI 1.0–20.4%) lower.

**Conclusions:**

Lower outdoor temperature was associated with adverse effects on cholesterol, blood pressure, circulating inflammatory markers, and vitamin D in two older populations. Public health approaches to protect the elderly against low temperatures could help in reducing the levels of several cardiovascular disease risk factors.

## Introduction

In the UK and most European countries, cardiovascular disease (CVD) risk increases at lower temperatures, a typical element of the cold season.^1,2^ As CVD risk during the cold season is more markedly increased in older rather than younger adults,^3^ investigating temperature-related variations in CVD risk factors in older adults is of particular interest.

It has been hypothesised that lower outdoor temperatures could exert their adverse effects by increasing the levels of well-established risk factors causally associated with coronary heart disease (CHD),^4,5^ such as blood pressure^6^ and lipids.^7^ However, associations of temperature with recently established causal risk factors for CHD, such as interleukin-6,^8^ are not well studied.^9^ Also, low outdoor temperatures may increase the levels of other novel risk factors prospectively associated with CVD (e.g. inflammatory markers, haemostatic markers),^10^ although the literature supporting this hypothesis is sparse.^9,11^ Higher outdoor temperature is also a proxy measure for sunlight exposure, and hence potentially related to the level of vitamin D which has consistently been associated with chronic disease incidence although its causal association remains hotly debated.^12^

Common limitations of previous studies investigating associations of outdoor temperature and CVD risk factors are small sample size,^13,14^ the specific geographical location,^11^ and the investigation of clinical populations.^15^ Therefore, large population-based studies which explore associations of outdoor temperature with a comprehensive range of CVD risk factors are required to improve statistical power and estimate precision.

Considering the gaps in knowledge from previous research, the aim of this study was to investigate the strength of relationship between established and novel biological risk factors and outdoor temperature in two large Northern European studies of older adults.

## Methods and participants

Participants from the British Regional Heart Study (BRHS) and the Prospective Study of Pravastatin in the Elderly at Risk (PROSPER) provided informed written consent, which was performed in accordance with the principles of the Declaration of Helsinki. The designs of BRHS and PROSPER, both prospective studies of cardiovascular disease comprising several thousand participants, have been previously described.^16^

### Cardiovascular risk factors measurement (outcomes)

For both BRHS and PROSPER, details of measurement values and classification methods for the cardiovascular risk factors were extensively described^16^ and are briefly reported here in the Supplementary Material, Cardiovascular Risk Factors Measurements. The measurements were carried out during 1997–2000, and the factors included (a) established risk factors, such as systolic and diastolic blood pressure (BP) obtained sitting, and blood lipids (triglycerides, total cholesterol, high density lipoprotein (HDL) cholesterol, and low density lipoprotein (LDL) cholesterol); and (b) novel risk factors, such as inflammatory factors (C-reactive protein (CRP), fibrinogen, interleukin 6 (IL-6)) and plasma viscosity (PV); haemostatic markers (tissue plasminogen activator (t-PA) antigen, fibrin D-dimer, von Willebrand factor (vWF); and vitamin D (VitD).

### Temperature data

National meteorological offices provided daily outdoor mean temperatures for the 24 towns of BRHS and three locations of PROSPER during the study period. Definition of outdoor mean temperature on the examination day (lag 0) has been extensively described elsewhere.^16^

## Statistical methods

### Descriptive statistics

Temperature and the unadjusted outcomes’ levels were examined by month of measurement. Then, excepting total cholesterol, *p*, HDL-cholesterol, LDL-cholesterol, systolic BP (SBP) and diastolic BP (DBP), all other outcomes were log-transformed for further analysis as their distributions were positively skewed.

### Associations (main effects) of temperature with the CVD risk factors

For log-transformed outcomes, associations were reported as the percentage change in the geometric mean associated with a decrease of 5℃ in mean temperature (5℃ being the standard deviation of daily mean temperature for the years 1997–2000 in the BRHS and PROSPER towns). Associations of temperature with BP variables, HDL-cholesterol, LDL-cholesterol, and total cholesterol, were reported as linear coefficients (absolute change) per decrease of 5℃ in mean temperature.

Before being considered for pooling, data from the BRHS and PROSPER were analysed separately due to differences in study design, inclusion criteria and measurement protocols. In BRHS, multilevel linear regression models (level 1 = individual, level 2 = town of examination) were used to take into account clustering within towns.^17^ Associations were adjusted for established CVD risk factors and possible confounders, such as age, body mass index (BMI), social class, smoking, alcohol consumption, physical activity score and time of day (measurement variable).^9^ In PROSPER, linear regression models were used to estimate the associations of temperature with the outcomes. Associations were adjusted for the same variables as for BRHS except for physical activity, social class, and time of day which were not ascertained, but for sex and location. In BRHS and PROSPER separately, the proportion of variance associated with temperature from the fully adjusted models was estimated using partial *R*-squared. We also fitted an interaction between temperature and age (both fitted as continuous variables) to test whether the relationship of temperature with outcomes was particularly marked among older participants.

### Pooled analysis

Regression coefficients from fully adjusted models of BRHS and PROSPER were pooled using a random effects model, to take account of heterogeneity between the two studies where it occurred, for each of the outcomes separately.

### Sensitivity analysis

The cumulative short-term effect of temperature on the CVD risk factors was also investigated, using the temperature moving average of seven days which included lag days from 0–6 prior to the examination day (lag 0–6).

An additional adjustment of outdoor temperature (at lag 0) with a seasonal term, such as day length or sine and cosine terms,^18^ was evaluated. However, since the variance inflation factor scores were between 9–13 for these seasonal terms when included with temperature, collinearity would have been induced and therefore the adjustment was not recommended in this case. Alternatively, we tested an adjustment of temperature with season fitted as binary variable (winter (December–March) vs summer (April–November)).

In BRHS, outdoor temperature was also additionally adjusted for indoor temperature (not available in PROSPER). As indoor temperature did not have any effect on the outcomes (all *p* > 0.05), and did not alter the magnitude of the associations of outdoor temperature with outcomes, it was not considered further.

As lung function measurement (forced expiratory volume in one second, or FEV_1_) was also available in the BRHS, a further sensitivity analysis was carried out adding FEV_1_ as covariate in models predicting CRP levels, to take into account the possible temperature-related variation in CRP due to poor respiratory health in winter.

## Results

### Participants

The BRHS and PROSPER participants’ characteristics are shown in Table 1. In BRHS, 4252 men out of 5516 survivors (77%) were examined during the study period. In PROSPER, 5804 participants out of 23,770 (24%) screened individuals participated in the clinical trial of pravastatin vs placebo. PROSPER participants were on average about seven years older than BRHS participants, with a higher percentage of never-smokers, and were less likely to drink alcohol (Table 1).

**Table 1.** The British Regional Heart Study (BRHS) and Prospective Study of Pravastatin in the Elderly at Risk (PROSPER) participant characteristics during examinations (1997–2000).

	BRHS men (*n* = 4252)^1^	PROSPER participants (*n* = 5804)^2^
**Demographic and background characteristics**		
Sex (male), *n* (%)	4252 (100)	2806 (48.0)
Age (years), mean (SD)	68.7 (5.5)	75.3 (3.3)
Social class (manual), *n* (%)	2166 (51.0)	–
**Physical health**		
Prevalence of stroke/ myocardial infarction, *n* (%)	370 (8.7)	979 (16.9)
Hypertension, *n* (%)	2703 (63.8)	3592 (61.9)
Diabetes, *n* (%)	380 (9.4)	623 (10.7)
BMI, mean (SD)	26.9 (3.7)	26.8 (4.1)
**Behavioural factors**		
*Smoking*		
Never, *n* (%)	1233 (29.1)	1969 (33.9)
Ex-smokers, *n* (%)	2464 (58.0)	2277 (39.2)
Smokers, *n* (%)	548 (12.9)	1558 (26.8)
*Alcohol consumption*		
None, *n* (%)	431 (10.3)	2576 (44.4)
Occasional/light, *n* (%) ^3^	2949 (70.5)	2698 (46.5)
Moderate/heavy, *n* (%) ^4^	779 (18.6)	530 (9.1)
Unclassified, *n* (%)	26 (0.6)	–
*Physical activity (PA) score*		
Inactive, *n* (%)	471 (11.5)	–
Occasional, *n* (%)	957 (23.4)	–
Light, *n* (%)	767 (18.7)	–
Moderate, *n* (%)	591 (14.4)	–
Moderate vigorous, *n* (%)	690 (16.8)	–
Vigorous, *n* (%)	621 (15.2)	–
**Biological markers, means (SD)**		
CRP, mg/l	3.53 (6.86)	5.94 (11.07)
IL-6, pg/ml	3.18 (2.95)	3.40 (3.08)
Fibrinogen, g/l	3.27 (0.74)	3.59 (0.74)
PV, mPa.s	1.285 (0.078)	1.296 (0.077)
t-PA, ng/ml	11.08 (4.44)	11.02 (4.04)
vWF, IU/dl	139.96 (46.19)	140.62 (45.98)
D-dimer, ng/ml	133.58 (210.74)	316.85 (189.48)
Tryglicerides, mmol/l	1.86 (1.08)	1.54 (0.74)
HDL-cholesterol, mmol/l	1.32 (0.34)	1.28 (0.36)
LDL-cholesterol, mmol/l	3.89 (0.97)	3.78 (0.83)
Total cholesterol, mmol/l	6.00 (1.08)	5.66 (0.94)
Vitamin D, ng/ml	20.01 (9.24)	16.57 (9.94)
SBP sitting, mm Hg	149 (24)	155 (22)
DBP sitting, mm Hg	85 (11)	84 (11)

BMI: body mass index; CRP: C-reactive protein; DBP: diastolic blood pressure; HDL: high density lipoprotein; IL-6: interleukin 6; LDL: low density lipoprotein; PV: plasma viscosity; SBP: systolic blood pressure; SD: standard deviation; t-PA: tissue plasminogen activator; vWF: von Willebrand factor.

1 BRHS men from England and Wales: *n* = 3804 (89.5%); from Scotland: *n* = 448 (10.5%).

2 Participants from Glasgow: *n* = 2520 (43.4%); from Cork: *n* = 2184 (37.6%), and from Leiden: *n* = 1100 (19.0%).

3 ≥1 and ≤15 units per week (one unit is approximately one drink, such as one glass of wine).

4 ≥16 units per week (one unit is approximately one drink, such as one glass of wine).

### Outdoor temperature of the day of examination by month

In both studies, daily mean temperatures on the day of examination were usually between 4–9℃ from November–April and between 10–16℃ from May–October (see Supplementary Material, eTable 1).

### CVD risk factors descriptive statistics by month

Highest levels of the CVD risk factors analysed were observed from November–April (see Supplementary Material, eTables 2–5). This variation was particularly marked for SBP, DBP, total cholesterol, CRP, IL-6, t-PA, vWF and PV. Conversely, VitD levels were lowest in colder months.

### Associations of temperature with the CVD risk factors

Adjusted associations of mean temperature on day of measurement with the CVD risk factors are shown in Table 2 for each study separately, and pooled.

**Table 2.** The change in the levels of cardiovascular disease (CVD) risk factors for a single standard deviation (5℃) decrease in outdoor mean temperature in the British Regional Heart Study (BRHS) and Prospective Study of Pravastatin in the Elderly at Risk (PROSPER) participants, during examinations (1997–2000).

					POOLED (BRHS + PROSPER)^c^		
	BRHS^a^		PROSPER^b^				Test of heterogeneity
	Percentage change (95% CI)	*p*-Value	Percentage change (95% CI)	*p*-Value	Percentage change (95% CI)	*I*^2^ (%)	*p*-Value
CRP, mg/l	4.1 (0.7–7.3)	0.017	2.4 (–0.8–5.7)	0.075	3.3 (1.0–5.6)	0.0	0.468
IL-6, pg/ml	1.8 (–1.3–4.8)	0.246	3.0 (1.1–4.9)	0.001	2.7 (1.1–4.3)	0.0	0.525
Fibrinogen, g/l	0.5 (–0.5–1.6)	0.286	0.8 (0.2–1.4)	0.007	0.7 (0.2–1.3)	0.0	0.677
t-PA, ng/ml	2.5 (0.6–4.4)	0.010	1.7 (0.6–2.8)	<0.001	1.9 (1.0–2.9)	40.6	0.461
PV, mPa.s	0.4 (0.2–0.6)	<0.001	0.4 (0.3–0.6)	<0.001	0.4 (0.3–0.5)	0.0	1.000
vWF, IU/dl	–1.0 (–2.6–0.7)	0.268	1.0 (0.0–2.0)	0.029	0.1 (–1.7–2.1)	74.0	0.050
D-dimer, ng/ml	1.6 (–1.5–4.6)	0.292	–0.6 (–2.1–0.9)	0.482	0.1 (–1.9–2.2)	0.0	0.516
Vitamin D, ng/ml	–6.1 (–9.1– –3.2)	<0.001	–16.0 (–17.5– –14.5)	<0.001	–11.2 (–20.4– –1.0)	97.3	<0.001
Triglycerides, mmol/l	1.5 (–0.7–3.6)	0.175	–0.1 (–1.3–1.1)	0.442	0.4 (–1.0–1.9)	39.3	0.199
	Absolute change (95% CI)	*p*-Value	Absolute change (95% CI)	*p*-Value	Absolute change (95% CI)	*I*^2^ (%)	*p*-Value
HDL-cholesterol, mmol/l	0.00 (–0.01–0.02)	0.844	0.03 (0.02–0.04)	<0.001	0.02 (–0.01–0.05)	90.6	0.001
LDL-cholesterol, mmol/l	0.05 (0.00–0.09)	0.039	0.02 (0.00–0.05)	0.038	0.03 (0.01–0.05)	0.0	0.346
Total cholesterol, mmol/l	0.06 (0.01–0.11)	0.015	0.04 (0.01–0.06)	0.004	0.04 (0.02–0.07)	0.0	0.464
SBP sitting, mm Hg	1.22 (0.29–2.16)	0.010	1.08 (0.45–1.71)	<0.001	1.12 (0.60–1.64)	0.0	0.796
DBP sitting, mm Hg	0.47 (0.04–0.90)	0.032	0.13 (–0.20–0.46)	0.270	0.27 (–0.06–0.60)	34.5	0.217

BMI: body mass index; CI: confidence interval; CRP: C-reactive protein; DBP: diastolic blood pressure; HDL: high density lipoprotein; IL-6: interleukin 6; LDL: low density lipoprotein; PV: plasma viscosity; SBP: systolic blood pressure; t-PA: tissue plasminogen activator; vWF: von Willebrand factor.

a Multilevel linear regression models (level 1 = individual, level 2 = town of examination) were used. The models were adjusted for age, social class, BMI, smoking, alcohol consumption, physical activity, and time of measurement. Complete case analysis (*n* = 3832)

b Linear regression models were used. The models were adjusted for town, age, BMI, smoking, alcohol consumption, and sex. Complete case analysis (*n* = 5804)

c Results from the two studies were pooled using a random effects model. The command *metan* with the option random available in Stata/SE 14. The percentage of variation across studies that is due to heterogeneity was reported using the *I*^2^ statistic.

Pooled estimates showed that with a 5℃ lower mean temperature, total cholesterol was 0.04 mmol/l (95% confidence interval (CI) 0.02–0.07) higher, LDL cholesterol was 0.02 mmol/l (95% CI 0.01–0.05) higher, and SBP was 1.12 mm Hg (95% CI 0.60–1.64) higher. Among novel CVD risk factors, CRP was 3.3% (95% CI 1.0–5.6%) higher, IL-6 was 2.7% (95% CI 1.1–4.3%) higher, t-PA was 1.9% (95% CI 1.0–2.9%) higher, fibrinogen was 0.7% (95% CI 0.2–1.3%) higher, and plasma viscosity was 0.4% (95% CI 0.3–0.5%) higher. There was no evidence of heterogeneity between studies (*p*-values > 0.05).

With a 5℃ lower mean temperature, VitD was 11.2% (95% CI 1.0–20.4%) lower. In this case, there was evidence of heterogeneity between studies (*I*^2 ^= 97.3%; *p*-value < 0.001), though the effect was in the same direction and statistically significant for both studies.

Associations of temperature with DBP, vWF and D-dimer, triglycerides, and HDL-cholesterol were not statistically significant. Results for HDL-cholesterol suggested heterogeneity (*I*^2 ^= 90.6%; *p*-value = 0.001) with association of a decrease in temperature significant for the PROSPER study only.

### Proportion of variance in risk factors explained by temperature

The highest proportion of variance was observed when the outcome analysed was VitD (5.1% and 5.6% in the BRHS and PROSPER fully adjusted models respectively). In each of the models, and other outcomes analysed, the proportion of variance associated with mean temperature was less than 1% (Supplementary Material, eTable 6).

### Interactions between temperature and age

Interaction effects of temperature with age on the outcomes levels were mainly not significant (data not shown). However, interactions were found in PROSPER alone for VitD and HDL-cholesterol. A 5℃ decrease in mean temperature was associated with an additional decrease of –0.8% per year of age (95% CI –1.4– –0.3%) for Vitamin D, and +0.003 mmol/l per year of age (95% CI 0–0.006) for HDL-cholesterol. No interactions were found in BRHS.

### Sensitivity analysis

Cumulative short-term associations of temperature up to one week (lag 0–6) prior to the examination day with the CVD risk factors levels were observed (not shown). As the magnitude of the associations was very similar to associations using temperature at lag 0 (primary analysis), only associations at lag 0 were presented.

An additional adjustment for season fitted as binary variable (winter vs summer) barely changed the magnitude of the associations of outdoor temperature (results were not shown).

In BRHS, an additional adjustment for lung function (FEV_1_) did not substantially change the effect of CRP: the percentage increase in CRP due to a decrease in temperature was 4.1% (0.7–7.3%) and 4.6% (1.4–7.8%) for models without and with lung function.

## Discussion

To our knowledge, the pooled analysis of the BRHS and PROSPER is the largest investigation of the relationships between outdoor temperature and an extensive range of CVD risk factors, both established and novel in older European people. The CVD risk factors investigated here were selected for two reasons: first, there was published evidence of seasonal variation, with higher levels observed in the cold season (November–April); second, there was published evidence of independent association with CVD events in meta-analyses of prospective population-based studies.^19–22^

### Overall findings

Lower outdoor temperature, measured on the day of clinical examination, was associated with higher levels of most CVD risk factors analysed. Conversely, lower outdoor temperature was associated with a lower VitD. The direction and magnitude of these associations were similar in comparison with other studies,^6,7,11^ and persisted after adjustment for classic risk factors such as age, BMI, smoking, alcohol consumption, and physical activity. The findings were similar when using the outdoor temperature moving average of seven days, which included lag days from 0–6 prior to the examination day (lag 0–6). In fully adjusted models, the proportion of variance in risk factors explained by temperature was much smaller than other risk factors, being around 1% of the total variance (except for VitD, where variance explained was approximately 5%). There was no consistent evidence of an interaction of temperature with age on the wide range of CVD risk factors analysed.

These findings would be consistent with the suggestions from previous studies that, in addition to established risk factors such as cholesterol^7^ and BP,^6^ circulating inflammatory markers,^9^ and VitD^23^ showed strong associations with outdoor temperature and may contribute to increased incidence of CVD in winter.^1^ The association of temperature with SBP, LDL-cholesterol and IL-6 levels may be particularly relevant, as previous trials and Mendelian randomization (MR) studies support their causal role in CHD risk.^4,5,24^

### Established CVD risk factors

In this study lower outdoor temperatures were significantly associated with higher levels of SBP consistently with previous findings.^25^ The association with DBP was weaker and non-significant. Seasonal variation in SBP was previously shown to be greater in older than in younger subjects (while DBP was similar), and highly significantly related to outdoor temperature.^26^

We found decrease in temperature was associated with increased total cholesterol and LDL-cholesterol, as previously reported.^27^ In our study, a decrease of about 10℃ in temperatures would be associated with an increase of 0.06 mmol/l in LDL-cholesterol. According to previous studies, this absolute increase in LDL-cholesterol leads to an increase of approximately 1% in CVD mortality risk.^4^ The importance of HDL-cholesterol as a marker of CHD risk has been emphasised through its inclusion in the Framingham Risk Score.^28^ When pooling results from the two studies, we found no clear association between temperature and HDL-cholesterol although a positive association was seen in PROSPER. Lastly, associations of temperature with triglycerides were not significant as observed in previous studies.^7^

### Novel CVD risk factors

A decrease in temperature was associated with increased circulating levels of markers of inflammation, such as IL-6, CRP, fibrinogen and plasma viscosity. The inflammatory hypothesis of CVD is currently being formally tested in randomized controlled trials (RCTs).^24^ To date, MR studies for IL-6 suggested a causal role in CHD, in contrast to null associations in MR studies for CRP and fibrinogen.^8^ Therefore, the findings on IL-6 are particularly important: in this study a decrease of about 10℃ in temperatures (difference between the coldest and warmest month, January–August) would be associated with an increase of 0.06 pg/ml in IL-6 levels. According to previous IL-6 observational data this absolute difference was associated to an increase of 4.5% in CVD deaths.^29^ This broad estimation is in line with previous studies which took place in the same years (1998–2007) and attributed to temperatures 7% of the winter mortality in England and Wales.^30^

It is also possible that an acute (or short-term) effect of outdoor temperature may be more marked on rapidly responding CVD risk factors, such as CRP.^31^ The CRP behaviour may explain why it provides closer associations and better predictions of CVD events in the short-term than other markers of inflammation. The associations of temperature with other specific markers of inflammation we studied, such as fibrinogen and plasma viscosity, were smaller in comparison with CRP, as previously reported.^15^

Findings for PV and t-PA are similar in comparison with previous studies which observed higher levels of these factors in winter,^9^ although the effect of temperature was not specifically tested. To our knowledge these associations with temperature are novel, and have not been previously published. On the other hand, the association of temperature with vWF and fibrin D-dimer was not significant. The seasonal variation in temperature did not show a good agreement with variations observed in vWF and D-dimer: vWF’s seasonal peak was previously observed in early spring (between March–May)^9^ when outdoor temperature already started its annual average increase from February; D-dimer seemed to have an unusual seasonal variation, with peaks in February/March and August/September.^32^

### VitD

Findings for VitD showed strong associations with temperature in pooled analysis, though this varied between the studies. However, for VitD specifically, temperature is likely to be a proxy of exposure to sunlight, which is the real determinant. In our study a decrease of about 10℃ in temperatures would be associated with a decrease of approximately 4 ng/ml (=10 nmol/l) in VitD levels. According to previous observational studies, this absolute decrease in VitD is associated with an increase of approximately 4% in CVD deaths and events^12^ although any causal role remains contentious.

### Strengths and limitations

By pooling PROSPER and BRHS we substantially improved statistical power and precision in comparison with findings reported in other studies of older adults. The participants lived mostly in the UK but also in Ireland and the Netherlands. The PROSPER study included both women and men. Moreover, novel CVD risk factors measurements in both studies were performed over the same time period, in the same Glasgow University laboratories using the same assays. However, the two study designs are different and this may partially explain the heterogeneity of the findings for HDL cholesterol, as well as the interactions of temperature with age; the PROSPER participants in comparison with the BRHS participants were about seven years older on average, with a higher percentage of never-smokers, and less likely to drink alcohol. They were also at elevated CVD risk and around half had prevalent CVD. Due to the nature of our data and risk of collinearity between temperature and other seasonal terms, it was not possible to distinguish between temperature-related effects and effects due to other factors which are known to vary by season: for example, in winter higher prevalence of influenza or other respiratory viruses or diseases, such as rheumatic disorders, may be relevant to the CRP seasonal variation. Despite this limitation, we took into account of season as binary variable (winter vs summer), and alternatively fitting respiratory health in sensitivity analysis: an additional adjustment for lung function was performed and specifically when using CRP as outcome. The results still showed that lower outdoor temperatures were significantly associated with an increase in the outcome levels. Moreover, although indoor temperature was not available in the PROSPER, we added this variable in the BRHS models and we showed the effect of outdoor temperatures was not confounded by indoor temperatures.

### Implications

Our study provides robust evidence that outdoor temperature is associated with variations in the major CVD risk factors in older adults. This study increased generalisability of existing evidence from northern European older populations and is consistent with the hypothesis that inflammation markers, on top of BP and LDL-cholesterol changes, could play a key role in intermediate processes leading to the cold-related CVD mortality. Also, there was no consistent evidence of an interaction of temperature with age (participants in the two studies ranged from 60–82 years old) on the wide range of CVD risk factors analysed; this finding suggested that the effect of low temperature on CVD risk may apply to the full age range of older adults. Public health approaches to protect elderly populations against low temperatures could help in reducing levels of several CVD risk factors, and thus CVD risk itself, in winter.

## Conclusions

Variations of outdoor temperature in the short-term were associated with variations in the majority of CVD risk factors analysed. Associations were strongest with inflammatory factors (particularly CRP, and its major cytokine driver, IL-6) and VitD, followed by associations with SBP, and cholesterol variables. Better protection against low temperatures could help in reducing the levels of several CVD risk factors.

## Supplementary Material

Supplementary material
