# Supplementary material for: Relationship between outdoor temperature and cardiovascular disease risk factors in older people
Source: Eur J Prev Cardiol. 2016 Nov 29;24(4):349–56. doi: 10.1177/2047487316682119 (PMC5301906; doi:10.1177/2047487316682119)
Supplement: Supplementary material [file CPR682119_Supplementary_Material.pdf]

Supplementary material

Cardiovascular Risk Factors Measurements

BRHS - 20-Year Examination (1998–2000)

Details of measurement values and classification methods for established and novel CVD risk factors, such as age, smoking status, social class, alcohol intake, physical activity, blood pressure (sitting systolic and diastolic), blood lipids, vitamins, haemostatic and inflammatory markers in this cohort were extensively described.[1-7] The participant's age was derived from the Q20 questionnaire's date. Specifically, physical activity levels were self-reported (SR) and method of assessment was described elsewhere.[8] The SR physical activity questionnaire was also recently validated using accelerometers.[9]

PROSPER - baseline (1997–1999)

Details of measurement values and classification methods for established and novel CVD risk factors, such as age, smoking status, alcohol intake, blood pressure (sitting systolic and diastolic), blood lipids, haemostatic and inflammatory markers in this cohort were extensively described.[10, 11] By study design, the participants plasma total cholesterol was required to be 4.0–9.0 mmol/L and their triglyceride concentrations less than 6.0 mmol/L; baseline levels were also reported elsewhere. [10, 12]

eTable 1 – Number of participants examined from the BRHS and PROSPER, total number of days when the examinations took place, and mean (SD) of daily average outdoor temperature during examinations (1997-2000). Note that for some months there are more days when examinations took place than days in the month, because measurements were taken across multiple years.

	BRHS (1998-2000)			PROSPER (1997-1999)		
Month	Number of BRHS men examined	Total number of days when the examinations took place	Daily mean temperature (SD) during examinations	Number of PROSPER participants examined	Total number of days when the examinations took place	Daily mean temperature (SD) during examinations
January	268	16	3.9 (2.4)	548	21	5.5 (2.7)
February	559	39	5.5 (3.4)	574	33	5.8 (2.9)
March	414	26	7.0 (2.5)	586	45	6.9 (2.7)
April	430	22	9.4 (2.7)	357	24	8.2 (2.7)
May	207	13	12.5 (2.4)	298	21	11.7 (2.3)
June	467	22	13.7 (2.3)	365	22	12.8 (2.3)
July	371	20	15.3 (2.3)	476	23	14.7 (1.2)
August	156	10	16.4 (2.0)	460	21	15.0 (1.8)
September	407	22	14.9 (2.2)	584	22	14.2 (2.1)
October	345	22	10.0 (2.2)	578	23	10.6 (2.7)
November	419	19	7.5 (2.5)	601	21	6.0 (3.1)
December	209	11	4.6 (2.2)	377	18	6.5(3.1)
Overall study period	4252	242	9.7 (4.8)	5804	294	9.8 (2.5)

eTable 2 – Means and standard deviations (in brackets) of CRP, IL-6, Fibrinogen, and PV by calendar month, and year in the BRHS and PROSPER participants, during examinations (1997-2000). Total number of participants by month and overall (n) is also reported.

	**CRP, mg/L**		**IL-6, pg/mL**		**Fibrinogen, g/L**		**PV, mPa.s**	
**Month**	**BRHS**	**PROSPER**	**BRHS**	**PROSPER**	**BRHS**	**PROSPER**	**BRHS**	**PROSPER**
January	3.20 (3.48)	6.33 (11.93)	3.53 (2.82)	3.49 (3.13)	3.27 (0.66)	3.59 (0.71)	1.298 (0.073)	1.305 (0.075)
	n=256	N=538	n=258	N=535	n=258	N=522	n=253	N=520
February	3.42 (6.07)	5.241(8.80)	3.12 (2.68)	3.29 (3.05)	3.25 (0.76)	3.60 (0.67)	1.285 (0.077)	1.304 (0.077)
	n=527	N=571	n=525	N=563	n=530	N=556	n=529	N=561
March	3.56 (8.75)	5.44 (8.04)	2.99 (3.10)	3.53 (3.06)	3.26 (0.82)	3.61 (0.69)	1.280 (0.086)	1.299 (0.079)
	n=405	N=572	n=405	N=570	n=405	N=550	n=378	N=556
April	3.27 (5.77)	7.156 (14.10)	3.36 (2.91)	3.65 (3.35)	3.22 (0.71)	3.70 (0.90)	1.276 (0.073)	1.295 (0.077)
	n=419	N=308	n=417	N=307	n=419	N=330	n=411	N=289
May	3.03 (4.29)	6.80 (17.83)	2.74 (2.16)	3.51 (3.37)	3.10 (0.60)	3.65 (0.88)	1.289 (0.104)	1.302 (0.082)
	n=192	N=296	n=194	N=295	n=194	N=285	n=188	N=290
June	3.66 (7.88)	6.29 (9.55)	2.83 (2.81)	3.49 (3.00)	3.18 (0.71)	3.69 (0.74)	1.285 (0.077)	1.290 (0.075)
	n=444	N=357	n=446	N=358	n=447	N=336	n=446	N=351
July	3.91 (7.76)	5.82 (16.03)	3.58 (3.00)	3.33 (3.07)	3.30 (0.72)	3.50 (0.74)	1.284 (0.080)	1.286 (0.078)
	n=353	N=466	n=351	N=466	n=356	N=435	n=353	N=451
August	3.22 (5.56)	5.22 (7.13)	2.72 (2.19)	3.37 (3.01)	3.39 (0.70)	3.57 (0.71)	1.292 (0.069)	1.290 (0.079)
	n=149	N=453	n=152	N=452	n=151	N=439	n=148	N=431
September	3.08 (7.40)	5.94 (9.86)	3.01 (2.84)	3.059 (2.684)	3.33 (0.68)	3.60 (0.73)	1.280 (0.072)	1.289 (0.070)
	n=389	N=579	n=386	N=577	n=392	N=563	n=385	N=533
October	4.19 (7.56)	5.72 (9.64)	3.50 (3.49)	2.98 (2.64)	3.35 (0.82)	3.47 (0.73)	1.292 (0.078)	1.292 (0.076)
	n=327	N=573	n=329	N=571	n=329	N=563	n=327	N=508
November	3.76 (6.90)	5.80 (9.48)	3.43 (3.48)	3.73 (3.48)	3.31 (0.69)	3.62 (0.69)	1.292 (0.071)	1.304 (0.079)
	n=397	N=592	n=398	N=588	n=399	N=583	n=396	N=574
December	3.80 (6.09)	6.78 (10.95)	3.03 (2.80)	3.58 (3.21)	3.36 (0.85)	3.58 (0.72)	1.284 (0.075)	1.290 (0.074)
	n=198	N=375	n=189	N=371	n=200	N=369	n=199	N=372
Year	3.53 (6.86)	5.94 (11.07)	3.18 (2.95)	3.40 (3.08)	3.27 (0.74)	3.59 (0.74)	1.285 (0.078)	1.296 (0.077)
	n=4056	N=5680	n=4050	N=5653	n=4080	N=5531	n=4013	N=5436

eTable 3 – Means and standard deviations (in brackets) of t-PA, PV, vWF, D-dimer by calendar month, and year in the BRHS and PROSPER participants, during examinations (1997-2000). Total number of participants by month and overall (n) is also reported.

	t-PA, ng/mL		vWF, IU/dL		D-dimer, ng/mL	
**Month**	**BRHS**	**PROSPER**	**BRHS**	**PROSPER**	**BRHS**	**PROSPER**
January	12.27 (5.24)	11.37 (3.86)	155.38 (52.34)	135.20 (43.02)	145.84 (212.44)	323.69 (192.50)
	n=258	N=515	n=258	N=515	n=258	N=525
February	11.49 (4.56)	11.27 (4.15)	136.30 (46.49)	139.88 (43.77)	125.89 (185.17)	297.40 (166.01)
	n=531	N=548	n=531	N=547	n=531	N=563
March	11.38 (4.18)	10.83 (4.05)	138.17 (45.95)	139.56 (44.13)	130.90 (238.78)	302.53 (179.41)
	n=405	N=552	n=405	N=546	n=405	N=560
April	11.69 (4.55)	11.93 (4.22)	135.15 (41.93)	148.55 (50.57)	141.68 (218.75)	310.91 (199.67)
	n=419	N=337	n=419	N=336	n=419	N=342
May	11.12 (4.43)	11.17 (4.32)	137.09 (45.83)	148.75 (51.04)	119.61 (156.62)	310.01 (188.79)
	n=194	N=284	n=194	N=282	n=193	N=290
June	10.58 (4.19)	11.69 (4.44)	129.47 (42.43)	141.01 (43.24)	117.94 (157.32)	339.08 (188.03)
	n=448	N=348	n=448	N=348	n=446	N=355
July	9.95 (4.29)	10.72 (4.21)	145.19 (46.70)	146.98 (55.68)	151.31 (266.53)	314.95 (178.90)
	n=356	N=449	n=356	N=447	n=356	N=457
August	11.33 (4.30)	10.93 (3.87)	144.86 (42.67)	137.85 (44.08)	116.24 (146.27)	323.37 (190.53)
	n=151	N=440	n=151	N=439	n=151	N=444
September	9.99 (4.12)	10.48 (3.63)	142.47 (47.74)	136.17 (44.80)	132.34 (177.53)	315.57 (197.03)
	n=392	N=548	n=392	N=546	n=391	N=565
October	11.49 (4.49)	10.93 (4.23)	136.03 (42.19)	135.62 (43.65)	132.30 (175.84)	327.15 (202.36)
	n=329	N=561	n=329	N=558	n=329	N=562
November	10.91 (4.14)	10.84 (3.88)	145.74 (49.52)	146.43 (45.06)	158.86 (306.42)	313.16 (179.77)
	n=400	N=576	n=400	N=576	n=400	N=584
December	11.27 (4.48)	10.53 (3.61)	146.76 (44.18)	137.79 (42.66)	110.44 (132.23)	334.62 (215.63)
	n=200	N=369	n=200	N=369	n=200	N=371
Year	11.08 (4.44)	11.02 (4.04)	139.96 (46.19)	140.62 (45.98)	133.58 (210.74)	316.85 (189.48)
	n=4083	N=5527	n=4083	N=5509	n=4079	N=5618

eTable 4 – Means and standard deviations (in brackets) of lipids levels by calendar month, and year in the BRHS and PROSPER participants, during examinations (1997-2000). Total number of participants by month and overall (n) is also reported.

	**Tryglicerides, mmol/L**		**HDL-cholesterol, mmol/L**		**LDL-cholesterol, mmol/L**		**Total cholesterol, mmol/L**	
**Month**	**BRHS**	**PROSPER**	**BRHS**	**PROSPER**	**BRHS**	**PROSPER**	**BRHS**	**PROSPER**
January	2.04 (1.03)	1.58 (0.75)	1.32 (0.34)	1.30 (0.36)	3.83 (1.06)	3.74 (0.81)	6.02 (1.20)	5.67 (0.93)
	n=251	N=542	n=248	N=542	n=244	N=542	n=251	N=542
February	1.80 (0.96)	1.56 (0.75)	1.33 (0.36)	1.31 (0.37)	3.82 (0.95)	3.83 (0.82)	5.92 (1.04)	5.76 (0.95)
	n=526	N=583	n=523	N=583	n=519	N=582	n=526	N=583
March	1.77 (1.03)	1.51 (0.75)	1.39 (0.37)	1.28 (0.37)	4.03 (0.96)	3.82 (0.85)	6.17 (1.07)	5.68 (0.96)
	n=401	N=576	n=395	N=576	n=394	N=575	n=401	N=576
April	1.82 (1.01)	1.51 (0.70)	1.26 (0.31)	1.26 (0.35)	4.10 (0.98)	3.92 (0.86)	6.15 (1.09)	5.74 (0.99)
	n=413	N=356	n=412	N=356	n=406	N=356	n=413	N=356
May	1.81 (1.23)	1.49 (0.69)	1.27 (0.33)	1.31 (0.36)	3.73 (0.96)	3.82 (0.85)	5.76 (1.06)	5.65 (0.97)
	n=192	N=304	n=191	N=304	n=191	N=304	n=192	N=304
June	1.78 (1.08)	1.57 (0.77)	1.33 (0.34)	1.27 (0.35)	3.91 (0.96)	3.79 (0.85)	5.97 (1.03)	5.66 (0.96)
	n=443	N=377	n=442	N=376	n=442	N=376	n=443	N=377
July	1.95 (1.10)	1.46 (0.73)	1.34 (0.35)	1.24 (0.33)	3.72 (1.03)	3.69 (0.80)	5.86 (1.11)	5.53 (0.89)
	n=351	N=480	n=343	N=480	n=340	N=480	n=351	N=480
August	1.69 (0.88)	1.57 (0.79)	1.37 (0.36)	1.23 (0.33)	3.82 (0.81)	3.74 (0.82)	5.90 (0.95)	5.66 (0.91)
	n=149	N=448	n=149	N=448	n=146	N=448	n=149	N=448
September	1.98 (1.46)	1.53 (0.72)	1.29 (0.32)	1.25 (0.34)	3.89 (0.97)	3.73 (0.80)	6.04 (1.09)	5.60 (0.91)
	n=384	N=584	n=383	N=584	n=380	N=584	n=384	N=584
October	1.88 (1.19)	1.58 (0.75)	1.33 (0.33)	1.30 (0.37)	3.96 (1.04)	3.80 (0.86)	6.07 (1.21)	5.69 (0.96)
	n=328	N=577	n=327	N=577	n=324	N=577	n=327	N=577
November	1.91 (0.97)	1.53 (0.72)	1.32 (0.33)	1.33 (0.37)	3.88 (0.91)	3.82 (0.80)	6.01 (1.02)	5.72 (0.90)
	n=395	N=604	n=394	N=604	n=392	N=604	n=395	N=604
December	1.83 (0.84)	1.49 (0.73)	1.36 (0.33)	1.30 (0.42)	3.87 (0.88)	3.66 (0.80)	6.01 (0.96)	5.58 (0.91)
	n=199	N=373	n=199	N=373	n=196	N=373	n=199	N=373
Year	1.86 (1.08)	1.54 (0.74)	1.32 (0.34)	1.28 (0.36)	3.89 (0.97)	3.78 (0.83)	6.00 (1.08)	5.66 (0.94)
	n=4032	N=5804	n=4006	N=5803	n=3974	N=5801	n=4031	N=5804

eTable 5 – Means and standard deviations (in brackets) of Vitamin D, SBP and DBP by calendar month, and year in the BRHS and PROSPER participants, during examinations (1997-2000). Total number of participants by month and overall (n) is also reported.

	Vitamin D, ng/mL		SBP sitting, mm Hg		DBP sitting, mm Hg	
**Month**	**BRHS**	**PROSPER**	**BRHS**	**PROSPER**	**BRHS**	**PROSPER**
January	14.50 (6.77)	12.57 (7.54)	150 (25)	156 (21)	86 (11)	84 (11)
	n=203	N=368	n=267	N=548	n=267	N=548
February	17.56 (7.20)	12.21 (7.02)	150 (24)	158 (21)	86 (12)	85 (11)
	n=496	N=379	n=558	N=574	n=558	N=574
March	17.55 (8.19)	13.46 (10.17)	150 (24)	156 (23)	86 (11)	84 (12)
	n=341	N=520	n=410	N=586	n=410	N=586
April	16.67 (7.76)	14.15 (11.53)	149 (23)	152 (22)	85 (11)	83 (12)
	n=383	N=541	n=426	N=357	n=426	N=357
May	18.11 (8.63)	14.78 (7.62)	146 (25)	155 (22)	83 (12)	84 (11)
	n=183	N=486	n=207	N=298	n=207	N=298
June	21.15 (8.58)	17.55 (8.44)	147 (23)	152 (22)	85 (11)	82 (11)
	n=439	N=494	n=466	N=365	n=466	N=365
July	20.63 (9.14)	19.706 (9.07)	148 (27)	151 (21)	84 (12)	83 (11)
	n=335	N=381	n=370	N=476	n=370	N=476
August	29.27 (9.92)	21.203 (9.20)	153 (24)	153 (22)	87 (11)	84 (12)
	n=142	N=513	n=155	N=460	n=155	N=460
September	24.28 (8.76)	20.28 (12.38)	146 (24)	155 (22)	84 (11)	84 (12)
	n=378	N=646	n=407	N=584	n=407	N=584
October	24.42 (11.24)	18.66 (9.20)	153 (23)	155 (22)	85 (11)	84 (11)
	n=324	N=476	n=344	N=578	n=344	N=578
November	19.53 (8.38)	16.28 (8.48)	149 (24)	155 (21)	84 (11)	84 (12)
	n=386	N=295	n=417	N=601	n=417	N=601
December	19.67 (9.40)	14.90 (8.16)	148 (24)	155 (21)	85 (11)	84 (11)
	n=189	N=274	n=208	N=377	n=208	N=377
Year	20.01 (9.24)	16.57 (9.94)	149 (24)	155 (22)	85 (11)	84 (11)
	n=3799	N=5373	n=4235	N=5804	n=4235	N=5804

eTable 6 – Total variance explained (Full adjusted models), and variance explained by temperature in Cardiovascular Risk Factors for the BHRS and PROSPER participants, during the study period (1997-2000)

BRHS	Total variance explained (%)	Variance explained by temperature (%)
CRP, mg/L	14.14	0.16
IL-6, pg/mL	17.70	0.03
Fibrinogen, g/L	9.95	0.08
t-PA, ng/mL	24.47	0.08
PV, mPa.s	8.54	0.31
vWF, IU/dL	10.58	0.04
D-dimer, ng/mL	14.18	0.01
Vitamin D, ng/mL	21.00	5.21
Tryglicerides, mmol/L	13.89	0.03
HDL-cholesterol, mmol/L	17.23	0.03
LDL-cholesterol, mmol/L	3.23	0.09
Total cholesterol, mmol/L	3.30	0.13
SBP sitting, mm Hg	6.09	0.27
DBP sitting, mm Hg	3.83	0.11

PROSPER	Total variance explained (%)	Variance explained by temperature (%)
CRP, mg/L	7.17	0.02
IL-6, pg/mL	6.48	0.14
Fibrinogen, g/L	4.39	0.09
t-PA, ng/mL	12.54	0.14
PV, mPa.s	5.34	0.42
vWF, IU/dL	6.45	0.05
D-dimer, ng/mL	5.12	-0.01
Vitamin D, ng/mL	15.01	5.59
Tryglicerides, mmol/L	8.86	-0.02
HDL-cholesterol, mmol/L	18.31	0.61
LDL-cholesterol, mmol/L	7.80	0.05
Total cholesterol, mmol/L	12.33	0.12
SBP sitting, mm Hg	2.58	0.17
DBP sitting, mm Hg	3.50	-0.01

References

1. Emberson JR, Whincup PH, Walker M, Thomas M and Alberti KG. Biochemical measures in a population-based study: effect of fasting duration and time of day. *Annals of clinical biochemistry*. 2002; 39: 493-501.
2. Emberson JR, Whincup PH, Morris RW, Walker M, Lowe GD and Rumley A. Extent of regression dilution for established and novel coronary risk factors: results from the British Regional Heart Study. *European journal of cardiovascular prevention and rehabilitation : official journal of the European Society of Cardiology, Working Groups on Epidemiology & Prevention and Cardiac Rehabilitation and Exercise Physiology*. 2004; 11: 125-34.
3. Wannamethee SG, Shaper AG, Lennon L and Whincup PH. Decreased muscle mass and increased central adiposity are independently related to mortality in older men. *Am J Clin Nutr*. 2007; 86: 1339-46.
4. Wannamethee SG, Shaper AG, Whincup PH, Lennon L and Sattar N. Obesity and risk of incident heart failure in older men with and without pre-existing coronary heart disease: does leptin have a role? *Journal of the American College of Cardiology*. 2011; 58: 1870-7.
5. Wannamethee SG, Tchernova J, Whincup P, et al. Plasma leptin: associations with metabolic, inflammatory and haemostatic risk factors for cardiovascular disease. *Atherosclerosis*. 2007; 191: 418-26.
6. Wannamethee SG, Welsh P, Papacosta O, Lennon L, Whincup PH and Sattar N. Elevated parathyroid hormone, but not vitamin D deficiency, is associated with increased risk of heart failure in older men with and without cardiovascular disease. *Circulation Heart failure*. 2014; 7: 732-9.
7. Wannamethee SG, Bruckdorfer KR, Shaper AG, Papacosta O, Lennon L and Whincup PH. Plasma vitamin C, but not vitamin E, is associated with reduced risk of heart failure in older men. *Circulation Heart failure*. 2013; 6: 647-54.
8. Wannamethee G and Shaper AG. Physical activity and stroke in British middle aged men. *BMJ : British Medical Journal*. 1992; 304: 597-601.
9. Jefferis BJ, Sartini C, Ash S, Lennon LT, Wannamethee SG and Whincup PH. Validity of questionnaire-based assessment of sedentary behaviour and physical activity in a population-based cohort of older men; comparisons with objectively measured physical activity data. *Int J Behav Nutr Phys Act*. 2016; 13: 14.
10. Shepherd J, Blauw GJ, Murphy MB, et al. Pravastatin in elderly individuals at risk of vascular disease (PROSPER): a randomised controlled trial. *The Lancet*. 2002; 360: 1623-30.
11. Stott DJ, Robertson M, Rumley A, et al. Activation of hemostasis and decline in cognitive function in older people. *Arteriosclerosis, thrombosis, and vascular biology*. 2010; 30: 605-11.
12. Lloyd SM, Stott DJ, de Craen AJ, et al. Long-term effects of statin treatment in elderly people: extended follow-up of the PROspective Study of Pravastatin in the Elderly at Risk (PROSPER). *PLoS One*. 2013; 8: e72642.
